# Impact of COVID-19 Pandemic on Patients With Neurodegenerative Diseases

**DOI:** 10.3389/fnagi.2021.664965

**Published:** 2021-04-08

**Authors:** Chao Hu, Cao Chen, Xiao-Ping Dong

**Affiliations:** ^1^State Key Laboratory for Infectious Disease Prevention and Control, NHC Key Laboratory of Medical Virology and Viral Diseases, Collaborative Innovation Center for Diagnosis and Treatment of Infectious Diseases (Zhejiang University), National Institute for Viral Disease Control and Prevention, Chinese Center for Disease Control and Prevention, Beijing, China; ^2^Center for Biosafety Mega-Science, Chinese Academy of Sciences, Wuhan, China; ^3^Center for Global Public Health, Chinese Center for Disease Control and Prevention, Beijing, China; ^4^China Academy of Chinese Medical Sciences, Beijing, China

**Keywords:** coronavirus disease 2019, severe acute respiratory syndrome-associated coronavirus 2, Parkinson’s disease, Alzheimer’s disease (AD), amyotrophic lateral sclerosis, Creutzfeldt-Jakob disease

## Abstract

COVID-19 pandemic has already produced great impacts on global health security and social-economy. Elderly, particularly those with underlying diseases, are suffering from higher fatality rate. Neurodegenerative diseases are a group of incurable neurological disorders of loss of neuron and/or myelin sheath, which affect hundreds of millions of elderly populations and usually need long-term care. Older population is one of the most vulnerable to COVID-19 pandemic. In this report, we reviewed the current status of COVID-19 on the patients with several neurodegenerative diseases, particularly Alzheimer’s disease, Parkinson’s disease, prion disease, and amyotrophic lateral sclerosis. Meanwhile, the potential mechanisms of SARS-CoV-2 infection in the pathogenesis of neurodegenerative diseases were also summarized.

## Introduction

As of March 12, 2021, coronavirus disease 2019 (COVID-19) has affected 224 countries, areas or territories, more than 118,000,000 confirmed cases and 2,600,000 deaths has been reported worldwide (WHO, 2021). The COVID-19 pandemic is disrupting the health systems and threatening the lives and health of people worldwide in an unprecedented way. Severe acute respiratory syndrome-associated coronavirus 2 (SARS-CoV-2), the causative agent of COVID-19, is able to trigger cytokine storm in infected individuals leading to malignant outcomes (Mehta et al., 2020). Although all populations are susceptible to SARS-CoV-2, COVID-19 causes obviously higher fatality rate among the elderly and individuals with weakened immune systems, especially those with underlying diseases (The Editors of Alzheimer’s & Dementia, 2020). Study have shown that SARS-CoV-2 can invade central nervous system (CNS) and further lead to neurological dysfunction in a significant proportion of affected patients, and individuals with COVID-19 can develop acute CNS-related symptoms (Mao et al., 2020). During the acute phase of SARS-CoV-2 infection, neurological symptoms occur in approximately 36% of cases, 25% of which can be attributed to direct involvement of CNS (Heneka et al., 2020). It is worth noting that the majority of patients with neurodegenerative diseases are elderly, hence such double effects make them more susceptible to COVID-19.

Although thousands of studies regarding to COVID-19 and SARS-CoV-2 have been published in the past year, the data of the impact of COVID-19 on neurodegenerative diseases is still limited. In this report, we analyzed and reviewed the current status and possible influencing factors of the patients with neurodegenerative diseases from different countries during the COVID-19 pandemic based on data from multiple channels, hoping to propose the potential direction of further research and concern.

## COVID-19 and Alzheimer’s Disease

Alzheimer’s disease (AD) is a common neurodegenerative disease mainly affecting the population of elderly. Clinically, it is mainly manifested as severe cognitive function and memory decline (Joe and Ringman, 2019). Pathologically, it is mainly caused by amyloid β (Aβ) deposition and neurofibrillary tangles (NFTs) in the brain (van der Kant et al., 2020). There are also neuron loss and inflammatory response observed in the relevant brain regions (Wilcock et al., 2011; Guo et al., 2014). About half of AD patients require home or professional long-term care (Perry, 2020). During the COVID-19 pandemic, the patients with AD appeared to be disproportionately affected. The New York Times reported that as many as 80% of COVID-19 deaths are in long-term care, meaning that more than a third of deaths are those affected with AD (Perry, 2020). Analyses revealed that certain characteristics of AD patients may increase the risk of SARS-CoV-2 infection. Patients with AD may not be able to follow the recommendations issued by public health authorities to reduce the transmission of SARS-CoV-2, including hand hygiene, covering mouth and nose when coughing, monitoring and reporting symptoms of COVID-19, maintaining physical distance from others, and self-isolation at home alone (Brown et al., 2020).

Another impact that cannot be ignored is that the COVID-19 pandemic is putting more stress on the mental and psychological health of AD patients. The investigation from Boutoleau’s group proposed for the first time that home confinement had a great effect on neuropsychiatric symptoms in the AD patients with lower baseline cognitive function during COVID-19 epidemic (Boutoleau-Bretonniere et al., 2020). They found that the increase of psychological stress would further accelerate the deterioration of cognitive function. The longer the confinement, the worse the symptoms. Such phenomenon may associate with the reduction of social contact and stimulation of physical activity during home confinement. In addition, the study found that the duration of confinement was significantly correlated not only with symptom severity, but also with their caregiver’s distress. They recommended that during the crisis such as COVID-19 pandemic, social services authorities should provide more supports to caregivers in order to help them to cope with their own social isolation and the neuropsychiatric changes of the patients they cared for.

Apolipoprotein E4 (APOE4), the most important susceptibility gene for AD, is genetically associated with the common late onset familial and sporadic AD (Rao et al., 1996). In European ancestral populations, the APOE4 homozygous genotype is associated with a significant risk of AD (Mayeux, 2003; Kuo et al., 2020). A recent observation found that the population with APOE4 homozygotes had a 2.2-fold higher COVID-19 infection rate and a 4.3-fold higher fatal rate than those of ApoE3 homozygous, with a high prevalence of APOE4 in the patients with severe COVID-19. Among the recruited population, about 2.36% of the participants with European ancestry were APOE4 homozygous, while among those of SARS-CoV-2 positive 5.13% were APOE4 homozygous, highlighting that the risk of SARS-CoV-2 infection was doubled for APOE4 homozygous individuals. But the study also suggested that APOE4 variants may not be an independent risk factor for severe COVID-19 infection. The weakness in AD patients themselves make them be vulnerable to SARS-CoV-2 infection and more likely be severe phenotype. Furthermore, since respiratory problems are common in most patients with advanced AD (Perry, 2020) and SARS-CoV-2 infection mainly affects the respiratory system, symptoms become more severe once AD patients are infected with SARS-CoV-2. However, it needs to be clarified that AD patients are at increased risk of SARS-CoV-2 infection, but that AD itself is not the direct cause for COVID-19 susceptibility. Preliminary studies have also showed that the variations in angiotensin-converting enzyme 2 (ACE2) gene, HLA gene, and ABO blood group gene on hosts and cells were also associated with susceptibility or severity of COVID-19 (Bourgonje et al., 2020; Kaiser, 2020; Ovsyannikova et al., 2020; Severe Covid et al., 2020).

Numerous studies have showed that virus invasion of brain tissue is associated with COVID-19 (Alquisiras-Burgos et al., 2020; Baig and Sanders, 2020; Lechien et al., 2020). The penetration of SARS-CoV-2 into cells is mediated by binding to ACE2, a cell surface receptor distributing mainly in the respiratory epithelium, vascular endothelium, kidney, small intestine, and brain (Donoghue et al., 2000; Hamming et al., 2004; Yan et al., 2020). Increasing evidence shows that SARS-CoV-2 may firstly invade peripheral nerve terminals, and then enter the CNS via a synaptic connection pathway (Fenrich et al., 2020). In mild and moderate COVID-19 cases, olfactory (85.6%) and gustatory (88.0%) dysfunctions were recorded frequently. About 11% of those patients developed anosmia prior to any other clinical symptoms (Lechien et al., 2020). ACE2 has been found to be highly expressed in nasal goblet and ciliated cells, which strongly indicates the possibility that SARS-CoV-2 may enter the human brain through olfactory nerves (Klingenstein et al., 2021). Another potential invading route for SARS-CoV-2 is probably via ventricular choroid plexus into cerebrospinal fluid (CSF) and brain (Abate et al., 2020). The high expression of ACE2 in lateral ventricular choroid plexus further increases the possibility of SARS-CoV-2 invading the CNS (Abate et al., 2020; Moriguchi et al., 2020). Recently, the SARS-CoV-2 was also detected by genomic sequencing in CSF sample from a 24-year-old male COVID-19 patient in Japan (Moriguchi et al., 2020). The invitation and infection of SARS-CoV-2 in the CNS may produce unpredictable effect on neurodegenerative diseases.

Recently, it has been hypothesized that SARS-CoV-2 infection may silently initiate or accelerate neurodegeneration. ACE2 expressing cells, such as neurons and glial cells, can be used as susceptible targets for SARS-CoV-2 infection (Zhou et al., 2020). SARS-CoV-2 can activate glial cells, induce pro-inflammatory state, and even cause severe innate immune response and sustained increase in cytokine levels. In addition, persistent SARS-CoV-2 infection can also induce neuroimmune responses (Ur and Verma, 2020). Retrospective studies have showed that corticosteroids were frequently used in the treatment of hospitalized COVID-19 patients, while it’s worth noting that inappropriate corticosteroid therapy produced adverse neuropsychiatric symptoms, affecting about 35% of COVID-19 patients, including cognitive and sleep disorders, delirium, hypomania, mania, depression, and psychosis (Troyer et al., 2020). As AD usually has long term of clinical course, the impact of COVID-19 on AD progression, either by direct virus infection or by inappropriate therapeutic deserves long term observation.

## COVID-19 and Parkinson’s Disease

Parkinson’s disease (PD) is the second largest neurodegenerative disease in the world. The main pathological features are the absence of dopaminergic neurons in the dense area of substantia nigra, and the presences of the inclusion bodies in residual neurons namely Lewy bodies (Homayoun, 2018). The main clinical manifestations are tremor, myotonia, tardiness and abnormal gait. However, some non-motor symptoms, including sleep disorder, hypoxia, anxiety, depression and cognitive disorders, are also important factors affecting the life quality of PD patients (Armstrong and Okun, 2020). During the COVID-19 pandemic, social measures of containment or mitigation significantly affected the lifestyle of PD individuals (Helmich and Bloem, 2020). This is because increased stress levels in COVID-19 outbreaks directly increase psychological stress, which can temporarily aggravate various motor symptoms, such as tremor, gait freezing or movement disorders (Macht et al., 2007; Zach et al., 2017a,b; Ehgoetz Martens et al., 2018; Helmich and Bloem, 2020). Increased stress induces an underlying low kinetic energy stiffness syndrome that causes dopaminergic cells to lose their energy rapidly in response to toxin. Meanwhile, reduction of physical exercise during COVID-19 pandemic may lead to an increased psychological stress, while lack of aerobic exercise is likely to worsen motor symptoms in PD patients (Helmich and Bloem, 2020). In addition, the neurophilic property of SARS-CoV-2 can cause inflammatory responses in CNS, and further worsen the pathology of PD.

Angiotensin-converting enzyme 2 is also highly expressed in dopaminergic neurons. Although the expression levels of ACE2 in the brain of PD patients is decreased due to degeneration and loss of dopaminergic neurons, there are still evidences that SARS-CoV-2 can invade CNS of PD patients and further aggravate the CNS injury and clinical situations (Achbani et al., 2020; Pavel et al., 2020; Victorino et al., 2020). Recently, the first case of encephalopathic complications in a 74-year-old PD patient infected with SARS-CoV-2 was reported (Filatov et al., 2020). Study showed that PD patients with older age (mean, 78.3 years) and longer disease duration (mean, 12.7 years) were particularly susceptible to COVID-19, with a case fatality rate as high as 40% (Antonini et al., 2020). However, the sample size of the study was small, and the conclusion needed further clarification of lager sample size. Nevertheless, older PD patients infected with SARS-CoV-2 have a significantly higher risk of death than the age-matched individuals who do not infected with PD. Whether the incidence of SARS-CoV-2 infection in PD patients increases or not remains unclear. A recent large cohort study of a relatively unselected patients with homogeneous PD has found that the risk, morbidity and mortality of COVID-19 in patients with mild to moderate PD do not differ from those in the general population (Fasano et al., 2020). Hainque et al. believed that the rapid recognition of COVID-19 in PD patients was quite challenged, because the common clinical manifestations of COVID-19, such as fatigue, anosmia, flushing or limb pain, were also non-motor PD signs. In addition, the exact severity of SARS-CoV-2 infection in PD patients remains to be further observed (Hainque and Grabli, 2020). Although it is unlikely that the SARS-CoV-2 can cause or aggravate PD, it has been reported that SARS-CoV-2 can aggravate certain motor and non-motor symptoms (Sulzer et al., 2020).

## COVID-19 and Prion Disease

Prion diseases are a group of rare transmissible neurodegenerative diseases usually have a long incubation period and short clinical duration (Saa et al., 2016). Pathologically, it can cause the loss of neurons in infected areas of the brain, formation of vacuoles and sponge-like lesions, and activation of astrocytes and microglia (Prusiner et al., 1998). Approximately 85% of the patients were sporadic Creutzfeldt-Jakob disease (CJD). Recently, a patient with suspected diagnosis of CJD was reported to be infected with SARS-CoV-2. The patient was a man in his 60s who was infected during family gathering. Soon, he developed neurodegenerative symptoms, including mutism, right hemiplegia, spontaneous multifocal myoclonus, lethargy, and restlessness. He died 2 months after onset. Examinations of EEG, MRI, CSF RT-QuIC, CSF 14-3-3, and tau protein highly indicated the diagnosis of sporadic CJD (sCJD) (Young et al., 2020). He displayed a fast progression and a short duration compared with majority of sCJD patients (Young et al., 2020). It has been noticed that COVID-19 can sometimes trigger unspecific inflammatory in CNS (Ellul et al., 2020; Pan et al., 2020; Paterson et al., 2020). Coincidentally, increased inflammatory response is also commonly documented in the brains of sCJD patients and numerous prion infected experimental animals at preclinical and terminal stage, including activate microglia and astrocytes with the release of proinflammatory cytokines, such as IL-1, IL-6, IL-12, and TNF-a (Xie et al., 2013; Aguzzi and Zhu, 2017; Ma et al., 2019; Chen C. et al., 2020; Chen J. et al., 2020). Whether the infection of COVID-19 can aggravate the brain inflammatory reactions and eventually accelerate the progression of human prion diseases needs further observation.

## COVID-19 and Amyotrophic Lateral Sclerosis

Amyotrophic lateral sclerosis (ALS, also known as motor neuron disease, MND) is a progressive and fatal neurodegenerative disease characterized with the degeneration of the motor neurons in brain and spinal cord (Hardiman et al., 2017; Owens, 2017). Clinically, ALS displays muscle weakness, spasm, respiratory failure, and communication disorders (Kiernan et al., 2011; Brown and Al-Chalabi, 2017). The incidence rate of ALS is about 2.76 per 100,000 people, with a prevalence of 9.62 cases in every 100,000 people worldwide (Xu et al., 2020). Similar to other neurodegenerative conditions, ALS is thought to be caused by a combination of genetic factors, environmental factors and aging-related dysfunction (Masrori and Van Damme, 2020). The diagnosis of ALS is mainly based on medical history, physical examination, electrodiagnostic testing (with needle-EMG) and neuroimaging (Masrori and Van Damme, 2020). COVID-19 has presented challenges to clinical care for ALS patients. COVID-19 pandemic also showed impact on ALS diagnosis and relevant studies, e.g., many clinical trials, as the diagnostic process and monitor safety and efficacy outcomes in clinical trials relied largely on face-to-face visits (Andrews et al., 2020). The COVID-19 pandemic has increased the need to telemedicine and technological devices, making it difficult to provide the best care for patients (De Marchi et al., 2020; Pinto et al., 2020). Recently, results of an internet-based questionnaires including self-perceived anxiety, depression, motor worsening, and changes in clinical care indicates that COVID-19 emergency and its management exert a significant impact on health status of ALS patients, particularly those in the early stages and more aggressive course of the disease (Cabona et al., 2021). Three patients without previous neurologic or autoimmune disorders who were diagnosed with myasthenia gravis after COVID-19 infection, indicating that COVID-19 infection may break immunologic self-tolerance (Restivo et al., 2020). So far, accurate data on SARS-CoV-2 infection in ALS patients are not available. Thereby, based on the impact of COVID-19 on clinical care, diagnosis and related experimental studies of ALS, the indirect impact of COVID-19 on ALS patients seems to be significant.

## Concluding Remark

Up to now, several routes through which COVID-19 affects central nerve system have been proposed, such as the direct infection of SARS-CoV-2 upon neuronal cells, vast inflammatory agents induced by severe systemic inflammation flooding into brains, respiratory failure associated brain ischemia, thrombosis and stroke, etc. (Verkhratsky et al., 2020). Psychological stress is frequently noticed in COVID-19 patients, medical staffs and general population (Fiest et al., 2021; Osimo et al., 2021; Saita et al., 2021). The global impact of the COVID-19 pandemic is unprecedented, with the entire population vulnerable possibly until COVID-19 vaccines are developed and widely available. Patients with neurodegenerative diseases need special attention as a special group. Although the size and data of current studies for the impacts of COVID-19 on neurodegenerative diseases are still limited, several potential impacts have been already proposed. First, as the elderly population with underlying diseases, the patients with some neurodegenerative diseases, such as AD, have already revealed significantly higher case fatality rate and more susceptible to SARS-CoV-2. Second, COVID-19 pandemic has already showed the great influences on the routine processes of diagnosis, treatment and daily care of the patients of neurodegenerative diseases, such as amyotrophic lateral sclerosis, which may have even more significant impacts in the future. Third, the immune storm and inflammatory response induced by SARS-CoV-2 infection seems to be able to increase the risk having more severe COVID-19 cases in the patients of neurodegenerative diseases, such as prion disease. Fourth, COVID-19 may accelerate the progression of neurodegenerative diseases, though the mechanisms remain unclear and may vary among different neurodegenerative diseases, such as AD and PD. Although different types of vaccines for COVID-19 are being vaccinated globally, when COVID-19 pandemic will slowdown, even stop, is still questionable. Therefore, the exact impacts of COVID-19 on neurodegenerative diseases need further long-term observation and investigation in order to propose appropriate interventions.

## Author Contributions

CH and CC designed the study and drafted the manuscript. CC and X-PD conceived the study, participated in its design, and drafted the manuscript. All authors contributed to the article and approved the submitted version.

## Conflict of Interest

The authors declare that the research was conducted in the absence of any commercial or financial relationships that could be construed as a potential conflict of interest.
